# MALDI-TOF MS Analysis of Cellodextrins and Xylo-oligosaccharides Produced by Hindgut Homogenates of *Reticulitermes santonensis*

**DOI:** 10.3390/molecules19044578

**Published:** 2014-04-11

**Authors:** Catherine Brasseur, Julien Bauwens, Cédric Tarayre, Christel Mattéotti, Philippe Thonart, Jacqueline Destain, Frédéric Francis, Eric Haubruge, Daniel Portetelle, Micheline Vandenbol, Jean-François Focant, Edwin De Pauw

**Affiliations:** 1 Mass Spectrometry Laboratory, Department of Chemistry, University of Liège, Allée du Six Aout, B6c, Sart-Tilman, B-4000 Liège, Belgium; E-Mail: e.depauw@ulg.ac.be; 2 Organic and Biological Analytical Chemistry, Department of Chemistry, University of Liège, Allée du Six Aout, B6c, Sart-Tilman, B-4000 Liège, Belgium; E-Mail: jf.focant@ulg.ac.be; 3 Department of Functional and Evolutionary Entomology, Gembloux Agro-Bio Tech, University of Liège, 2 Passage des Déportés, B-5030 Gembloux, Belgium; E-Mails: julien.bauwens@ulg.ac.be (J.B.); frederic.francis@ulg.ac.be (F.F.); e.haubruge@ulg.ac.be (E.H.); 4 Department of Bio-Industries, Gembloux Agro-Bio Tech, University of Liège, 2 Passage des Déportés, B-5030 Gembloux, Belgium; E-Mails: cedric.tarayre@ulg.ac.be (C.T.); p.thonart@ulg.ac.be (P.T.); j.destain@ulg.ac.be (J.D.); 5 Department of Microbiology and Genomics, Gembloux Agro-Bio Tech, University of Liège, 6 Avenue Maréchal Juin, B-5030 Gembloux, Belgium; E-Mails: matteochris@netscape.be (C.M.); daniel.portetelle@ulg.ac.be (D.P.); m.vandenbol@ulg.ac.be (M.V.)

**Keywords:** xylo-oligosaccharides, cellodextrins, termites, MALDI-TOF MS, *Trichoderma reesei*

## Abstract

Hindgut homogenates of the termite *Reticulitermes santonensis* were incubated with carboxymethyl cellulose (CMC), crystalline celluloses or xylan substrates. Hydrolysates were analyzed with matrix-assisted laser desorption/ionization coupled to time-of-flight mass spectrometry (MALDI-TOF MS). The method was first set up using acid hydrolysis analysis to characterize non-enzymatic profiles. Commercial enzymes of *Trichoderma reesei* or *T. longibrachiatum* were also tested to validate the enzymatic hydrolysis analysis. For CMC hydrolysis, data processing and visual display were optimized to obtain comprehensive profiles and allow rapid comparison and evaluation of enzymatic selectivity, according to the number of substituents of each hydrolysis product. Oligosaccharides with degrees of polymerization (DPs) ranging from three to 12 were measured from CMC and the enzymatic selectivity was demonstrated. Neutral and acidic xylo-oligosaccharides with DPs ranging from three to 11 were measured from xylan substrate. These results are of interest for lignocellulose biomass valorization and demonstrated the potential of termites and their symbiotic microbiota as a source of interesting enzymes for oligosaccharides production.

## 1. Introduction

Lignocellulosic biomass is increasingly considered as a renewable feedstock for fuels and chemicals, with the advantages of low production costs, availability and no competition with food or feed production. The structural polysaccharides of plant cell walls, the cellulose and hemicellulose, represent a major source of oligosaccharides and monosaccharides for potential production of bioethanol and biochemicals. The tight association between lignin, hemicellulose and cellulose is responsible for the recalcitrance of lignocellulosic biomass and remains the most important barrier to improve sugar recovery and biomass valorization. Termites, well-known as wood feeders and symbiotic hosts, represent a potential source of efficient lignocellulose-degrading enzymes to isolate [1,2]. They are also a very interesting model to study; a better understanding of how the termite ecosystem degrades lignocellulose may help to improve industrial enzymatic digestion processes and lead to novel biotechnological applications [3,4].

The enzymatic cellulose degradation is achieved by the cellulase complex, consisting of endo-1,4-β-glucanases (EC 3.2.1.4), exoglucanases including cellobiohydrolases (EC 3.2.1.91) and cello-dextrinases (EC 3.2.1.74), and β-glucosidases (EC 3.2.1.21). A summary of substrates and specific hydrolysis products is available in Table 1. Most studies on termites evaluated the cellulolytic activity in their digestive system with reducing ends analysis based on colorimetric detection [5,6,7]. This method evaluates how efficiently an enzymatic system is able to hydrolyze cellulosic substrates. It is, however, not specific with regard to the fragments produced. The analysis of intermediate degradation products is important when studying a cellulolytic activity, as some compounds can be involved in retro-inhibition mechanisms [8,9], can undergo post cleavage modification like transglycosylation or phosphorylation [10,11], or can induce enzyme gene expression [12]. Cellodextrins are β,1-4 glucose oligomers that are produced during cellulose hydrolysis. Their analysis is of interest not only to investigate hydrolysis mechanisms, but also because they possess valuable properties for microbial utilization and health care purposes [13]. Up to date, the high cost and low productivity of cellodextrins have restrained their applications.

**Table 1:** Overview of the cellulase complex specificities.

Cellulase Activity	Substrate	Hydrolysis Products
Endo-1,4-β-glucanase	Amorphous cellulose, soluble cellulose	Glucose, cellobiose, cellotriose, higher oligomers
Cellobiohydrolase	Crystalline cellulose, soluble cellodextrins	Glucose, cellobiose
Cellodextrinase	Soluble cellodextrins	Glucose, cellobiose
β-1,4-glucosidase	Cellobiose, soluble cellodextrins	Glucose

Some other interesting oligosaccharides are the xylo-oligosaccharides (XOS) produced from xylan, a main constituent of hemicelluloses. Xylan consists in a backbone of xyloses, often acetylated or branched with arabinose and acidic sugars, with various compositions. Xylan degradation requires a large set of enzymes including xylanases (EC 3.2.1.8), β-xylosidases (EC 3.2.1.37), α-arabinofuranosidase (EC 3.2.1.55), α-glucuronidase (EC 3.2.1.139) and acetylxylan esterase (EC 3.1.1.72). Studies on hemicellulose degradation by termites are less common, although this plays an important role to enhance enzyme accessibility to the cellulose trapped in a lignocellulose substrate [14]. However, hydrolysis mechanisms need further investigation as some XOS negative effect on cellulase activity was reported [15,16]. XOS also possess interesting prebiotic properties and their potential for medical, food and health care applications have been already demonstrated [17]. Industrial production of XOS is achieved by thermochemical and enzymatic degradation of lignocellulosic biomass rich in xylan, such as hardwood like beechwood or birchwood. For food or pharmaceutical industries, enzymatic hydrolysis production of XOS has advantages over autohydrolysis production to prevent undesirable side reactions and products. Interesting enzymes could be isolated from termites and their symbiotic microbiota to improve the lignocellulosic biomass valorization regarding its potential for oligosaccharides production.

In this study, matrix-assisted laser desorption/ionization coupled to time-of-flight mass spectrometry (MALDI-TOF MS) was used to enable direct analysis of the composition of hydrolysates. MALDI-TOF MS is a suitable method for fast and simple determination of the molecular weight of oligosaccharides, especially with a high degree of polymerization (DP). High-performance liquid chromatography coupled to refractive index detection (HPLC-RI) or high-performance anion-exchange chromatography coupled to pulsed amperometric detection (HPAEC-PAD) are alternative methods for oligosaccharides detection that allow quantitative analysis but they present some disadvantages. HPLC-RI is limited for detection of oligosaccharides with DP higher than six [18]. HPAEC-PAD allows detection of oligosaccharides with wide DP ranges but in the case of XOS derived from hemicellulose in lignocellulosic biomass, the method is limited due to their low solubility at room temperature or from the various compositions of side chains and substituents. These factors also influence the chromatographic separation [18]. Mass spectrometry analysis improves the identification of oligosaccharides mixture, allows detection of potentially modified products and provides additional information concerning substituents in derivative substrates. MALDI-TOF MS has been already used for chemical structural characterization of cellulose derivatives, and especially for determining the degree of substitution of the substrate [19,20,21]. In the case of enzymatic degradation, the number of substituents on each produced fragment can be correlated to enzymatic selectivity. This has previously been reported for purified endoglucanases issued from efficient cellulolytic organisms like *Trichoderma reesei* and *Humicola insolens* [22,23,24]. When investigating enzyme selectivity or hydrolysis mechanisms, the ability to form specific products is more valuable than the amount of products formed.

Oligosaccharides released from carboxymethyl-cellulose (CMC), crystalline celluloses and xylan degradation were analyzed. CMC is a derivative soluble substrate that has been preferably used in termite studies to evaluate endo-1,4-β-glucanase activity. Crystalline cellulosic substrates were also tested in the hope to have a better evaluation of endoglucanase activity on naturally occurring cellulose. The hemicellulolytic activity was evaluated with xylan substrate. The aim of the study was to analyze the intermediate oligosaccharides formed during the hydrolysis process by termites and bring additional information to provide keys for greener fuels and chemical production from lignocellulosic biomass.

## 2. Results and Discussion

### 2.1. Oligosaccharides Produced from Cellulose Derivative

**Figure 1:**
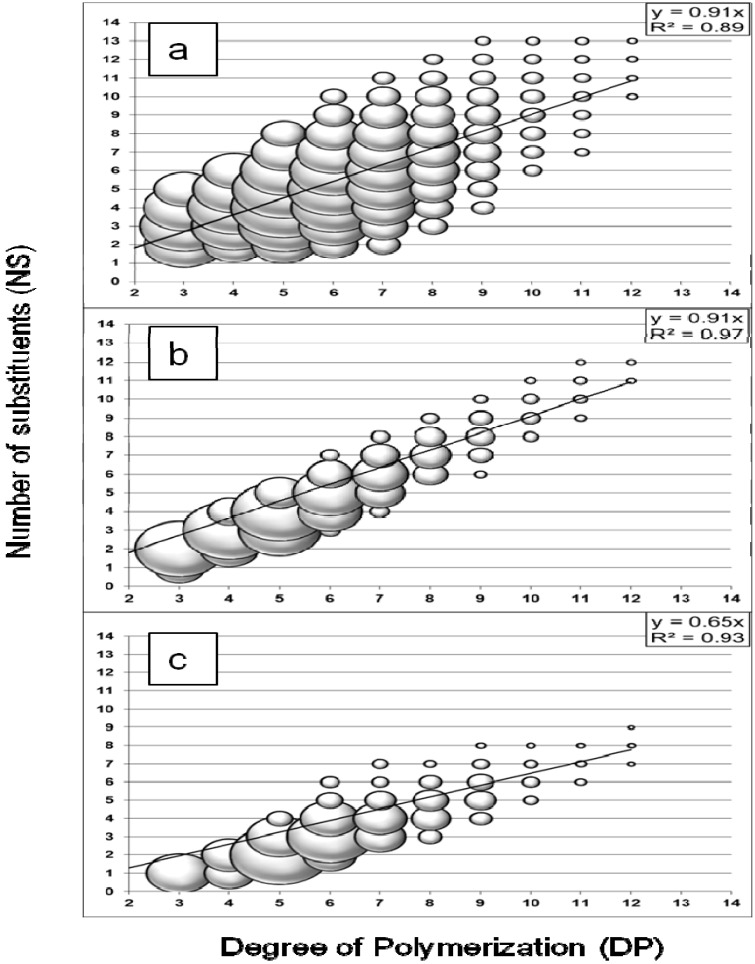
Comparison of carboxymethyl cellulose (CMC) hydrolysis profiles obtained with (**a**) trifluoroacetic acid (TFA) 2M, (**b**) a commercial cellulase from *Trichoderma reesei*, (**c**) termite hindgut homogenates (*Reticulitermes santonensis*). The oligosaccharides produced were analyzed by MALDI-TOF MS and displayed with markers according to their degree of polymerization (DP) and total number of substituents (NS), identified from *m/z* values. The relative intensity of each compound was represented by the spherical volume of the marker.

The acid hydrolysis of CMC was analyzed to characterize a non-enzymatic profile. As a random depolymerization was expected, it was also carried out to evaluate the ionization efficiency of the hydrolysis products because no substituted standard compounds were available. In the case of derivative cellulose substrate, a discrimination against molecules with few substituents was reported for MALDI-TOF MS analysis of CMC [21]. The acid hydrolysis profile of CMC (Figure 1a) showed a large distribution of oligosaccharides with DPs from three to 12 and an average number of substituents (NS) range, with DP-dependency, from NS = DP − 5 to NS = DP + 3. The results demonstrated no significant discrimination against oligomers with few substituents as they were detected with relative intensity (represented by the spheric volume of the markers) similar to oligomers with the highest number of substituents. However, the intensity decreased for oligomers with DP higher than eight because of the discrimination against larger oligomers for MALDI analysis in positive mode [21]. It was suggested that the cationisation process from adduct formation favors the smaller molecules, which are likely more soluble and able to migrate to the surface of the sample spot and generate a signal. The relationship between DP and NS values was demonstrated with a linear regression model (least squares method), with R^2^ = 0.89 and a slope value of 0.91. In the case of acid hydrolysis, this provided an interesting method to calculate the degree of substitution (DS) of CMC *i.e.* the average number of carboxymethyl substituent per unit of glucose. An average similar DS value (0.88 ± 0.02, RSD 2.5%, n = 4) was calculated with the method (DS_Level Off_) proposed by Enebro and Karlsson [25]. This method (measuring DS where the curve levels off) was based on the fact that the difference between the measured and the true DS should decrease with increasing DP, considering the lower ionization efficiency for oligomers with few substituents discussed by Momcilovic *et al.* [21]. However, the estimated values calculated with both methods remained higher than the DS value of 0.7 specified by the supplier of the CMC used in this study. Overestimation when measuring the DS in CMC by MALDI-TOF MS was also discussed by Enebro and Karlsson [25]. Additionally to the influence of the cationisation process dependent on the total number of substituents, they suggested an influence of the homogenization of the sample spot and the crystal size as they obtained better estimation with spectra acquisition centered in the target spot. In this study, part of the overestimation of DS could be explained because of the automated random shooting of the entire spot applied for all analyses.

The method was validated for the analysis of enzymatic hydrolysis using a commercial *T. reesei* cellulase (Figure 1b). The DPs of the measured oligosaccharides ranged from three to twelve and the NS values were also DP-dependent, but within a limited range from NS = DP − 2 to NS = DP + 1, on average. Additionally, a higher relative intensity was obtained for some compounds. This preferential formation of hydrolysis products reflected the selectivity of an enzymatic system, for which the hydrolysis reaction was limited by the presence of substituents on the substrate, probably from steric hindrance at the enzyme’s active site. A linear correlation was also demonstrated with a higher coefficient of determination (R^2^ = 0.97). The slope value was similar to the acid hydrolysis result (0.91). 

The hydrolysis profile obtained with the termite hindgut homogenates and CMC substrate is shown in Figure 1c. The profile presented the characteristics of an enzymatic hydrolysis profile: the NS range for similar DP was limited to a few values and some compounds presented higher relative intensity. The measured oligosaccharides had DPs from three to twelve with a NS range from NS = DP − 5 to NS = DP − 2, on average. The relationship between DP and NS values was linear (R^2^ = 0.93) but the slope value (0.65) was lower than for the other profiles.

**Figure 2:**
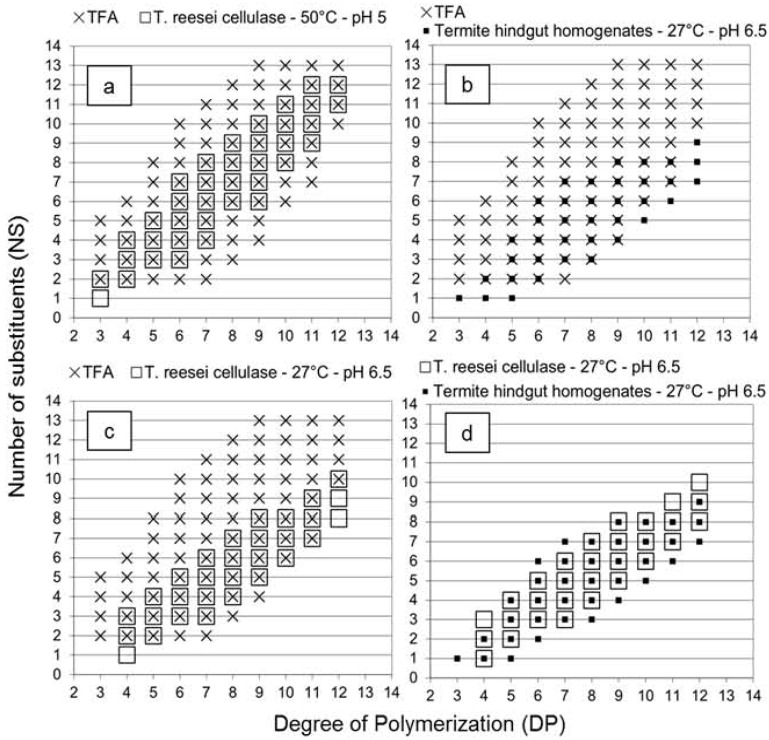
The enzyme selectivity of the *Trichoderma reesei* cellulase (empty squares) and the termite hindgut homogenates (black squares) can be evaluated based on the number of substituents (NS) obtained for each degree of polymerization (DP) of the oligosaccharides produced from carboxymethyl cellulose (CMC) hydrolysis. The *T. reesei* cellulase was tested with different conditions of pH and temperature and trifluoroacetic acid (TFA) 2 M was used for comparison with non-enzymatic hydrolysis reference profile (**a**–**d**). The oligosaccharides were analyzed by MALDI-TOF MS.

It is interesting to note that similar slope values were obtained for acid hydrolysis and *T. reesei* cellulase profiles, despite the enzymatic selectivity of the *T. reesei* cellulase. The explanation was clearly demonstrated in Figure 2 with the overlay of the different graphs. Oligomers with fewer substituents were not detected in the *T. reesei* cellulase profile in comparison with the acid hydrolysis profile (Figure 2a). In the termite profile, oligomers with fewer NS values similar to the acid hydrolysis profile were detected (Figure 2b), and as the enzymatic selectivity inhibited production of oligomers with high NS value, the slope value of the termite profile was lower (0.65), as expected.

The high slope value of the *T. reesei* cellulase profile suggested an exoglucanase or β-glucosidase activity on oligomers produced by the endoglucanase activity (see Table 1). Enzymes were likely able to find and hydrolyze one or two unsubstituted glucose units at the extremity of the produced oligomers, resulting from the selective endoglucanase activity. Consequently, the DP value of each oligomer decreased of one or two units while the NS value remained constant, increasing the slope of the linear relationship between DP and NS values. This additional activity influenced the distribution of the oligomers produced and compensated the decrease of the slope value due to the endoglucanase selectivity. Results obtained with the *T. reesei* cellulase at 27 °C and pH 6.5 (Figure 2c) supported this hypothesis, as oligomers with fewer NS values similar to the acid hydrolysis profile were detected and a lower slope was obtained. This suggested that a temperature of 27 °C and a pH value of 6.5 were not optimal conditions for the exoglucanase or β-glucosidase activity observed. Therefore, without the influence of these activities on the *T. reesei* profile, Figure 2d demonstrates that the *T. reesei* cellulase and the termite hindgut homogenates presented similar enzymatic selectivity at a temperature of 27 °C and a pH value of 6.5, although the comparison should be evaluated carefully as enzyme powder and a complex microbiota system were compared. However, these results opened several perspectives to study the parameters influencing the enzymatic selectivity, to evaluate predominant activities in termites from comparison with isolated symbiont profiles, as well as to evaluate endoglucanase activity and potential enzymes for cellodextrins production in cellulolytic systems.

### 2.2. Oligosaccharides Produced from Crystalline Cellulose

The analytical method was evaluated with standard compounds available for glucose and different cellodextrins: cellobiose, cellotriose and cellotetraose (Sigma-Aldrich). Glucose and cellobiose were not detected below mM levels, due to matrix interferences and limited ionization efficiency. The instrumental limits of detection (iLODs) for cellotriose and cellotetraose were estimated to be 3.5 µM and 1 µM, respectively, based on a signal-to-noise ratio of 3. Analysis of a mixture of cellotriose and cellotetraose at similar levels demonstrated the higher ionization efficiency for cellotetraose, with a signal 3 times higher than cellotriose. These results were important for the analysis of enzymatic profiles, to properly evaluate higher intensity obtained for some DP oligomers.

**Figure 3:**
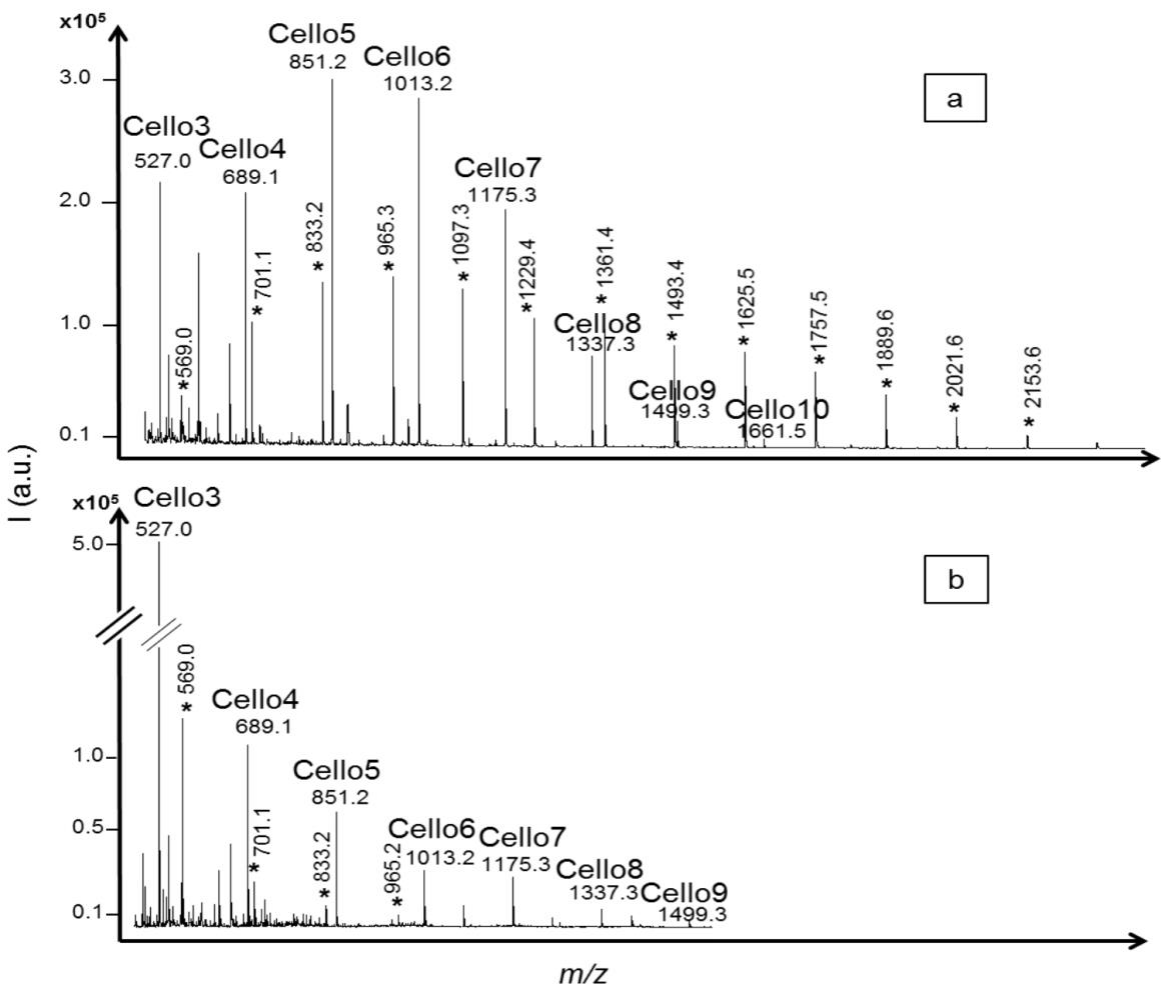
MALDI mass spectra of cellodextrins produced from α-cellulose hydrolysis by (**a**) trifluoroacetic acid (TFA) 2M; and (**b**) a commercial cellulase from *Trichoderma reesei*. Degradation products of interfering xylan included in the α-cellulose substrate were also detected and identified as xylo-oligosaccharides based on their *m/z* values (from 569.0 * to 2153.6 * corresponding to four to 16 xylose units including one sodium adduct).

Avicel and α-cellulose substrates were both tested to compare the influence of the crystallinity index on the hydrolysis reaction. A previous study reported crystallinity indexes for the commercial substrates used in this project, with values 15 to 30% lower for α-cellulose, depending on the method used [26]. Acid hydrolysis as well as enzymatic hydrolysis with the *T. reesei* cellulase were analyzed to validate the method. Fewer cellodextrins (DPs from three to six) were detected using the Avicel substrate in both acid and *T. reesei* cellulase profiles, demonstrating that the crystalline structure reduced hydrolysis reaction. Cellodextrins with DPs from three to 10 (with one sodium adduct) were significantly detected (S/N > 10) in acid or *T. reesei* cellulase profiles using α-cellulose substrate (Figure 3). In the acid hydrolysis profile, the intensities of cellodextrins with DPs from three to seven were similar while the intensity of cellodextrins with DP higher than eight decreased, reflecting the discrimination against larger oligomers due to lower solubility. In the *T. reesei* cellulase profile, the highest signal intensity was obtained for cellotriose, around five times higher than cellotetraose, despite lower ionization efficiency demonstrated with standards. Cellotriose has already been reported as an abundant and important intermediate product in enzymatic hydrolysis mechanisms [27]. It is one of the most soluble cellodextrins [28] and its higher accumulation level, compared to cellotetraose, could be explained by its number of glucose units and potentially a lack of completely filling the number of active sites of endoglucanases for further cleavage [29]. A similar hydrolysis profile was obtained at a temperature of 27 °C and a pH value of 6.5. These conditions were characterized as not optimal for exoglucanase activity according to results obtained with CMC substrate. The similar profiles suggested that the results could be related mainly to endoglucanase activity.

Hydrolysis products with *m/z* corresponding to oligosaccharides of pentose units were also detected in the α-cellulose hydrolysis profiles. Extracted α-cellulose was reported to contain a percentage of xylan [30,31]. Xylo-oligosaccharides (XOS) with similar intensities were detected in the acid hydrolysis profile (Figure 3a, marked with *), from *m/z* = 569.0 to 2153.6, corresponding to four to 16 xylose units with one sodium adduct. XOS with DPs from four to seven were detected in the *T. reesei* cellulase profile, indicated as *m/z* = 569.0 to 965.2 (Figure 3b, marked with *). This demonstrated the advantages of the method in differentiating the products formed, in comparison with non-specific analysis like reducing ends analysis that would have normalized all the compounds as “glucose production”.

Hydrolyses of crystalline cellulose substrates were tested with the termite hindgut homogenates. Although hydrolysis profiles were obtained with CMC, no cellodextrins were detected by MALDI-TOF MS, even if the partially amorphous alpha-cellulose substrate was used or incubated with freshly prepared hindgut homogenates. The results obtained with the *T. reesei* cellulase (Figure 3b) clearly demonstrated the possibility of specifically evaluating endo-1,4-β-glucanase activity against crystalline cellulose with the method. It is possible that the endoglucanase activity from the hindgut homogenates was combined with higher activity of cellodextrinases and β-1,4-glucosidases, resulting in a limited accumulation of cellodextrins. However, no intermediate compounds were detected when experiments were performed with 1, 2, 4 or 6 h of incubation time with substrates. Levels were probably below detection limits. The cellulolytic system in lower termites like *R. santonensis* is described with endogenous secretion of endo-1,4-β-glucanases and β-glucosidases in the salivary glands [4]. These enzymes are therefore secreted physically earlier in the digestive tube, and they probably act primarily against the amorphous cellulose of the wood particles ingested by termites. However, amorphous cellulose represents a small part of the total amount of cellulose in wood and further digestion of the abundant crystalline cellulose is achieved in the symbiontic section of the gut, essentially by protistan cellobiohydrolases. In this study, the results suggested no significant endo-1,4-β-glucanase activity against crystalline cellulose in the symbiotic microbiota gut part of the termite *R. santonensis*. This was demonstrated with crystalline or partially amorphous non derivative cellulose substrates, more representative of naturally occurring cellulose than the conventional CMC substrate. The discovery of a mechanism of depolymerization of crystalline cellulose through endoglucanase activity would have been be interesting to improve lignocellulose valorization with cellodextrins production. More studies on cellulolytic organisms should be performed in that direction, according to the abundance of crystalline cellulose in lignocellulose and the need to improve enzymatic production of cellodextrins.

### 2.3. Oligosaccharides Produced from Xylan

**Figure 4:**
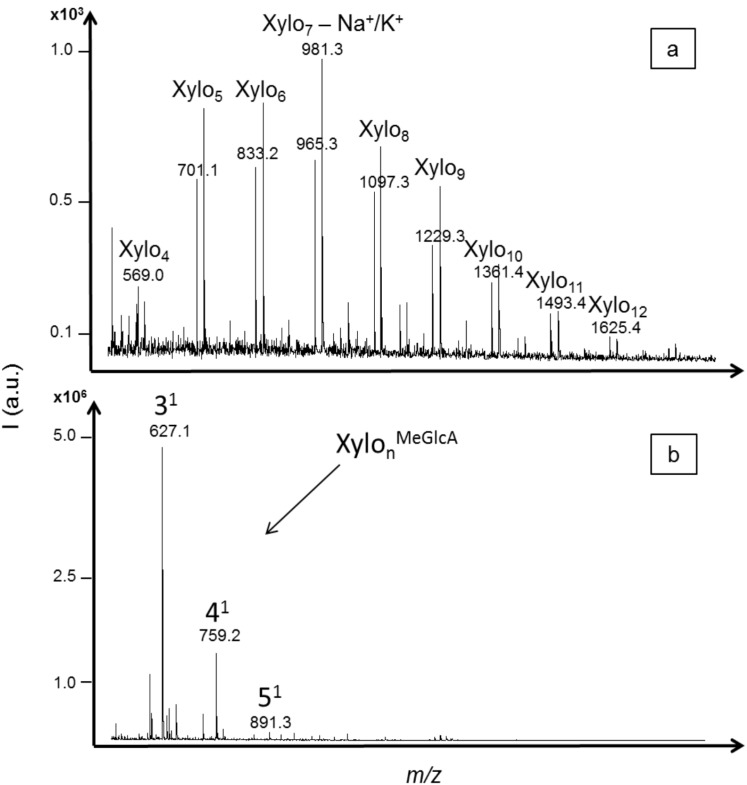
MALDI mass spectra of xylo-oligosaccharides (XOS) produced from xylan hydrolysis by (**a**) trifluoroacetic acid (TFA) 2 M; and (**b**) a commercial endo-1,4-xylanase from *Trichoderma longibrachiatum*. Neutral XOS were identified from acid hydrolysis based on their *m/z* values including one sodium or one potassium adduct. Acidic XOS corresponding to xylose units branched with one methylglucuronic acid were identified from enzymatic hydrolysis based on their *m/z* values including one sodium adduct.

Xylanase activity was evaluated using xylan substrate from birchwood. In hardwood, the principal hemicellulose is a backbone of xylose residues with 4-*O*-methylglucuronic acid substituent attached at various positions along the polysaccharide chain. Only neutral xylo-oligosaccharides with DPs ranging from four to twelve (with one sodium or one potassium adduct) were significantly detected (S/N > 10) with the acid hydrolysis (Figure 4a). These results showed that methylglucuronic side chains were hydrolyzed with acid treatment. Similar intensities were obtained for XOS with DPs from five to nine while the intensities of XOS with DP higher than 10 decreased. Also, a lower intensity was obtained for xylotetraose.

The enzymatic hydrolysis analysis was tested with an endo-1,4-beta-xylanase of *Trichoderma longibrachiatum*. Only acidic XOS were detected, with *m/z* values corresponding to oligomers of three to five xylose units carrying one methylglucuronic acid as side chain (including one sodium adduct) (Figure 4b). The aldotetrauronic acid (4-*O*-methylglucuronic acid linked to xylotriose) presented the highest intensity, five times higher than the aldopentauronic acid (4-*O*-methylglucuronic acid linked to xylotetraose). In this enzymatic hydrolysis profile, neutral XOS were not detected, probably because they were further hydrolyzed by the endoxylanase activity or by other additional enzymatic activities also reported for this commercial enzyme. The mode of action of endoxylanases belonging to the glycosyl hydrolase families (GHFs) 10 and 11, the two main families of endoxylanases, were characterized on acidic XOS [32]. It was demonstrated that neither aldotetrauronic acid nor aldopentauronic acid were substrates for endoxylanases of these families. The higher intensity obtained for the aldotetrauronic acid compound in the enzymatic profile supported the hypothesis of additional xylanase activity like β-xylosidase [33].

**Figure 5:**
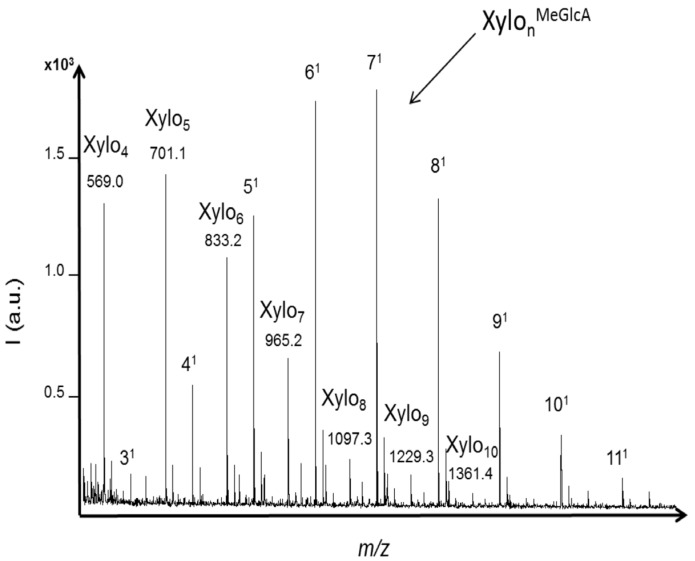
MALDI mass spectra of xylo-oligosaccharides (XOS) produced from xylan hydrolysis by termite hindgut homogenates. Both neutral and acidic XOS were identified based on their *m/z* values including one sodium adduct. Acidic XOS from three to eleven xylose units carrying one methylglucuronic acid as side chain were detected within the *m/z* range from 627.1 to 1683.5.

The enzymatic activity against xylan was evaluated with the termite hindgut homogenates. Both neutral and acidic XOS were detected in the profile (Figure 5). XOS with DPs ranging from four to 10 (with one sodium adduct) were significantly detected (S/N > 10) and the highest intensity was obtained for XOS with DPs from four to six. Acidic oligomers of three to 11 xylose units carrying one methylglucuronic acid as side chain were detected within the *m/z* range from 627.1 to 1683.5 (including one sodium adduct). The highest intensity was obtained for the acidic XOS with DPs from five to eight. The profile that was obtained was in accordance with the distribution of 4-*O*-methylglucuronic acid in xylan from birchwood that have been characterized as heterogenic and every 10 to 15 xylose residues on average [34,35]. The hydrolysis was likely achieved preferentially in regions with no substituted xylose residues, as 4-*O*-methylglucuronic acid substituents limited the action of endoxylanases. Then, further hydrolysis was probably achieved between glycosidic linkages closed to substituents, producing both neutral and acidic XOS. It was reported that endoxylanases of GHF-10 are able to cleave linkages in the xylan backbone closer to the substituents. Also, in the case of glucuronoxylan, the shortest acidic fragment released by GHF-10 endoxylanases was aldotetrauronic acid while GHF-11 released aldopentauronic acid. The detection of aldotetrauronic acid in the termite profile suggested that the result of GHF-10 endoxylanase activity could be detected in the profile, although GHF-10 xylanases were reported as much less abundant than GHF-11 in termites from the same family of *R. santonensis*, *i.e.* the *Rhinotermitidae* [4].

Most of the studies on xylanases in termites available to date are based on molecular genetic analysis or xylanolytic activity of isolated and purified enzymes evaluated with reducing ends analysis. Investigations with isolated xylanolytic bacteria and yeasts were also carried out [36]. It was interesting to evaluate the xylanase activity of a termite gut homogenate as the evaluation of purified enzymes or cultures could largely differ from the activities under natural conditions in the gut [2]. Moreover, the analysis of intermediate hydrolysis products is important in the case of xylan degradation, as xylan is the main source of oligosaccharides production during lignocellulosic biomass pretreatment. In our study, the MALDI-TOF MS method used was quite sensitive to allow the evaluation of XOS produced from xylan substrate by the equivalent of one termite hindgut. The results demonstrated the mechanism of depolymerization of xylan through endoxylanase, with production of intermediate XOS with DP up to 10.

Xylanolytic activity might play an important role in the efficient lignocellulose degradation achieved by the symbiont-termite association. It can probably be seen as a complementary activity to the mechanical pre-treatment of the wood achieved by the termite host, allowing the access to crystalline cellulose. More studies are needed on the addition of xylanases to cellulase mixtures to enhance enzyme accessibility and improve sugar recovery [14]. Inhibition mechanisms also need to be further investigated as well as the XOS responsible as different inhibitory effects on cellobiohydrolases were observed. It is possible that cellobiohydrolases produced by termites or their symbiotic microbiota are less inhibited by XOS or not inhibited by XOS with higher DP. In such a case, a better control of the DP of the produced oligosaccharides would be required, according to specific endoxylanases, amounts and conditions.

## 3. Experimental

### 3.1. Termites

*Reticulitermes santonensis* were collected on Oleron Island, France. The culture was maintained in a laboratory on wet wood at 27 °C and 70% humidity. Only mature workers were selected for experiments. Sets of ten hindguts pulled out of the termites were homogenized using a piston pellet (Eppendorf, Nijmegen, The Netherlands) in 0.5 mL of phosphate-buffered saline solution containing protease inhibitors (Roche, Brussels, Belgium). The termite hindgut homogenates were either used directly for hydrolysate preparation or kept at −80 °C until use.

### 3.2. Hydrolysate Preparation

Carboxymethyl cellulose (DS ~0.7), Avicel PH-101, α-cellulose and xylan (from birch wood) were purchased from Sigma-Aldrich (St. Louis, MO, USA). Hydrolysis were performed in 1.5-mL plastic tubes, with 20 mg of substrate mixed with 950 µL of 50 mM ammonium acetate buffer (pH 6.5) and incubated with 50 µL of termite hindgut homogenate (one hindgut equivalent) for 24 h at 27 °C using a thermomixer (Eppendorf, Nijmegen, The Netherlands). Temperature and pH were tested according to culture conditions and physiological termite gut pH values [37]. Significant and stable profiles were obtained with 24 h of incubation time. For cellulose hydrolysis reference profiles, equivalent of 40 µg of cellulase powder from *Trichoderma reesei* (ATCC 26921, ≥6 units/mg, defined by Sigma-Aldrich) were used for enzymatic hydrolysis at pH 5.0 and 50 °C for 24 h, and trifluoroacetic acid (TFA) 2 M was used for acid hydrolysis at 90 °C for 4 h. For xylan hydrolysis reference profiles, equivalent of 200 µg of endo-1,4-β-xylanase powder from *Trichoderma longibrachiatum* (Sigma ref X2629, ≥1 unit/mg, defined by Sigma-Aldrich) was used at pH 5.0 and 30 °C for 24 h. Controls without enzyme, termite homogenate or substrate were all included. Each condition was tested a minimum of three different times and with independent termite homogenate preparations. For each experiment, hydrolysates were prepared in triplicate and stored at −20 °C before analysis.

### 3.3. MALDI-TOF MS Analysis

Matrix 2,5-dihydroxybenzoic acid (DHB) was used at 20 mg/mL in a mixture of acetonitrile/water (v/v:50/50) with 0.1% trifluoroacetic acid. For CMC hydrolysate analysis, DHB was mixed with ammonium sulphate at 10 mg/mL (v/v:10/1) to reduce H^+^ /Na^+^ exchange with carboxymethyl groups that could produce *m/z* values that would interfere with data processing [25]. Samples were prepared with a mixture of 1 µL of hydrolysate solution and 1 µL of matrix. For each experiment, the triplicated hydrolysates were spotted five times each, and spectra were added to take into account biological and analytical variations. Measurements were performed with the time-of-flight mass spectrometer Ultraflex II TOF/TOF (BrukerDaltonics, Bremen, Germany) equipped with a frequency-tripled Nd:YAG laser (355 nm). The spectra were recorded in positive reflectron ion mode, with an accelerating voltage of 25 kV and a laser shot rate of 100 Hz. The voltages of the electrodes 1 and 2 were set at 21.8 kV and 9.5 kV respectively, to carry out the pulsed ion extraction. The time delay before the ion extraction was set at 30 ns. A total of 10,000 shots were accumulated for each mass spectrum. Except for specific tests, the acquisition *m/z* range started at 500 to exclude high intensity signals from matrix ions. Calibration was performed with peptide calibration standard II solution (Bruker).

### 3.4. Data Processing

The acquired spectra were processed using FlexAnalysis software (Bruker Daltonics) to select mass peaks with a signal-to-noise ratio (S/N) higher than 3. The resulting data list was then exported to an Excel file to search for masses corresponding to hydrolysis products using an embedded reference table. For CMC, *m/z* values of potential hydrolysis products were calculated according to the degree of polymerization (DP), with one to three potential carboxymethyl substituents for each glucose unit and one sodium adduct. Matching *m/z* values were represented by markers on a graph according to the corresponding DP and total number of substituents (NS). A spherical volume was used for each marker to represent the intensity value of the detected compound. The intensity values were normalized relatively to the most abundant compound set to 100%. The hydrolysis profiles were also analyzed without the intensity dimension in order to overlay the graphs for a better comparison of the enzymatic selectivity, based on the NS values obtained for each DP.

## 4. Conclusions

CMC substrate is usually dedicated to evaluating endo-1,4-β-glucanases, more active on soluble cellulose than on crystalline cellulose. Crystalline cellulose substrate is usually dedicated to evaluating cellobiohydrolases, more active on crystalline cellulose than on CMC. This evaluation is relevant when using the reducing ends analysis, including glucose and cellobiose detection (see Table 1). In this study, the MALDI-TOF MS method was shown suitable to detect cellulose degradation products with DPs higher than three, which are produced by endo-1,4-β-glucanase activity. This was particularly interesting for screening potential efficient endoglucanases able to act on CMC as well as on crystalline celluloses. Also, cellodextrinase and β-1,4-glucosidase activity against the soluble cellodextrins produced were indirectly evaluated.

An interesting xylanase activity was demonstrated with the method. Termites, and especially *Reticulitermes* species, should be better considered for their potential for XOS enzymatic production. Xylanases purified from culturable termite gut bacteria are interesting for industrial applications. Two recent studies reported a bacterial GHF-11 xylanase and a xylanolytic *Bacillus subtilis* culture isolated from *R. santonensis* [38,39]. Most studies on termites have focused on efficient cellulose hydrolysis enzymes for fermentable sugars production but we demonstrated that termites could also be further investigated as a valuable source of enzymes for oligosaccharides production.
